# Diseases of the Nervous System

**Published:** 1906-02-24

**Authors:** 


### Progress in Medicine and Surgery.

### DISEASES OF THE NERVOUS SYSTEM.

**Cerebral Tumour.**—In studying tumours of the brain from the point of view of possible operation, it is seen that the views of surgeons vary widely. Thus, while Seidel gives the proportion of operable cases as 3 per cent., Dana places it as high as 17 per cent. This discrepancy is doubtless in some measure to be explained, as Walton and Paul [1] suggest, by the personal equation of writers on the subject. The last-named writers have made a study of 424 cases of cerebral tumour in which autopsies were performed, and, dividing the cases into three classes, the operable, the inoperable, and the doubtful, they conclude that 7 per cent. were operable, 80 per cent. were inoperable, and 13 per cent. were doubtfully operable. Those tumours designated operable were such that removal would have offered a reasonable chance of cure, being primary, accessible, well-defined tumours, springing usually from the dura mater and coming under the heading of endotheliomata. Growths involving the deeper cerebral structures, the basal ganglia, the pituitary body or middle lobe of cerebellum, infiltrating gliomata, multiple growths and metastases are usually considered unsuitable for operation, while the doubtful class, the class presumably most affected by the personal equation, consists of gliomata and nonencapsulated superficial sarcomata, many subtentorial tumours, and cysts of which the contents can merely be evacuated. A number of operable growths failed to show localising symptoms, being situated near the median line or under the frontal or temporo-sphenoidal lobes. For this reason the authors condemn exploratory puncture, seeing that deep encapsulated tumours are rarely found post-mortem, and advocate rather a more extended method of search over the surface of the brain. The infrequency of the post-mortem diagnosis gumma is noteworthy in recent reports, a fact which contrasts strongly with the frequency with which the clinical diagnosis of this form of growth is made, especially where multiple symptoms are present. The authors, therefore, make the practical deduction that such a conclusion should not deter operation in suitable cases of intracranial growth. It must, however, be remembered that when gumma is diagnosed specific treatment is adopted and that therefore many cases never come to autopsy. The difficulty of accurate localisation without, or even with, definite symptoms, is forcibly illustrated by a case of brain tumour recorded by F.R. Fry.[2] The patient was a sluggish-minded countryman aged forty-three, described as "not bright." The general symptoms of intracranial tumour, double optic neuritis, headache, vomiting, and vertigo occurring in typical exacerbations, were well marked. The mental condition varied, at times he was very stupid and somnolent. Hearing and speech were unaffected. The gait was of the typical staggering cerebellar type, and the patient invariably veered to the left in walking, and frequently showed a tendency to stumble and fall to that side, the left leg often gave way, and in rising from a chair he would lunge to the left, or sometimes fall to the ground on that side. There was no hemiplegia or spasm, but the right foot sometimes shuffled and dragged. Tremor and nystagmus were absent, and also static ataxia, but the patient was clumsy and uncertain in the use of both arms, and at times some dysmetria, a jerkiness and lack of precision in movement sometimes observed in cerebellar cases, was noted. The right arm was used rather more than the left; but there was thought to be present a slight degree of hypertonia on that side, coupled with the cerebellar symptom of atonia on the left—cerebellar paresis as it is called. The condition of the reflexes varied as they may in tumour of the cerebellum, and, never very marked, could sometimes only be obtained by reinforcement. They were not decidedly stronger on the right side. Finally, the "screwing in" position of the head, the occiput turned to the left shoulder, and the chin slightly elevated and turned to the right, was frequently assumed, suggesting a lesion in the left cerebellar lobe. Although, as Dr. Grainger Stewart has pointed out, the "screwing in" or "screwing out" position of the head is not pathognomonic of cerebellar lesions and is occasionally observable in neoplasms of other parts of the brain, taken in conjunction with the other symptoms it was regarded as supporting the diagnosis made of tumour on the left side of the cerebellum. In accordance with this view the left side was thoroughly explored, but no tumour was found. The patient died five weeks later with cerebral œdema. At the autopsy a sharply defined nonencapsulated tumour, the shape of an orange, two inches in diameter, was found occupying the greater part of the situation of the left inferior frontal convolution and compressing the precentral and first and second temporal convolutions and those of the island of Reil. Subsequent inquiry elicited the fact that the patient had always been decidedly left handed. Although no structural changes were found in the cerebellum, Dr. Fry raises the question whether the symptoms so characteristic of disease of that organ may not have been produced by its having become crowded down by pressure into the foramen magnum in the manner already described by Dr. James Collier. Walton and Paul studied some of the symptoms also in their collection of 424 cases. Headache with vomiting was recorded in 91 cases, headache alone in 65, vomiting alone in 11 cases only. Convulsions accompanied tumours in all situations, but were most frequent in frontal growths. As others have already observed, they were not found to be of localising value, even when of the Jacksonian type, if appearing late in the illness. The reflexes were studied as carefully as the records permitted. The superficial reflexes were lessened in more than half the cases in which they were noted; a fact which is considered to bear out the supposition of Grasset that in man the controlling centre for such has risen to the cortex. The deep reflexes varied, but were found oftener increased than decreased. This again is thought to favour Grasset's view that the controlling mechanism of the deep reflexes has only ascended to the basal ganglia, for the chance of total destruction of the reflex arcs is less than that of interference with the inhibiting mechanism, which is presumably at a higher level. While unwilling to dogmatise, these writers urge a thoughtful reconsideration of the hypothesis that loss of knee-jerk can only be produced by damage to the spinal segments. The location of the reflex mechanism is also touched upon by Walton and Barrett[3] in a case of tumour of the pituitary body reported by them in which there were no symptoms of acromegaly. The knee-jerks were absent. Sections of the cord showed changes in the vicinity of the third cervical segment, in the region between the columns of Goll and Burdach, the so-called *Zwischen-zone* of Bechkren. A similar degeneration at this spot has been demonstrated by Reichardt in cases of general paralysis with Argyll-Robertson pupil. It is suggested that injury of afferent tracts of conduction may play a part in this loss and, at any rate, such a case tends to support Grasset's views of cerebral rather than lumbar location of the dominant centres presiding over the reflexes. Franceschi[4] describes a case of cerebral tumour marked by an excessive tendency to true sleep. A sarcoma was found in such a situation that it was closely surrounded by the circle of Willis, the arteries forming which were very greatly reduced in size. The case is interesting in the light it throws on the theories of sleep, of which the best-known ascribes it to anæmia of the cortex cerebri. Such a case is also not inconsistent with the theory of the existence of a sleep centre located in the central grey substance of the brain.

[1] Jour. of Nerv. and Ment. Dis., Aug. [2] Ibid., October. [3] Ibid., Aug., p. 531. [4] Ibid., Sept., p. 603.

